# Experiences of personal protective equipment by Australian healthcare workers during the COVID-19 pandemic, 2020: A cross-sectional study

**DOI:** 10.1371/journal.pone.0269484

**Published:** 2022-06-07

**Authors:** Darshini Ayton, Sze-Ee Soh, Danielle Berkovic, Catriona Parker, Kathryn Yu, Damian Honeyman, Rameesh Manocha, Raina MacIntyre, Michelle Ananda-Rajah

**Affiliations:** 1 Health and Social Care Unit, School of Public Health and Preventive Medicine, Monash University, Melbourne, Victoria, Australia; 2 School of Primary and Allied Health Care, Monash University, Melbourne, Victoria, Australia; 3 Monash-Cabrini Department of Musculoskeletal Health and Clinical Epidemiology, Melbourne, Victoria, Australia; 4 General Medical Unit, Alfred Health, Melbourne, Victoria, Australia; 5 Central Clinical School Monash University, Melbourne, Victoria, Australia; 6 Public Health Unit, Sydney Local Health District, New South Wales Health, Melbourne, Victoria, Australia; 7 HealthEd, Melbourne, Victoria, Australia; 8 Kirby Institute, University of New South Wales, Melbourne, Victoria, Australia; USM Advanced Medical and Dental Institute: Universiti Sains Malaysia Institut Perubatan dan Pengigian Termaju, MALAYSIA

## Abstract

The aim of this study was to capture Australian frontline healthcare workers’ (HCWs) experiences with personal protective equipment (PPE) during the COVID-19 pandemic in 2020. This was a cross-sectional study using an online survey consisting of five domains: demographics; self-assessment of COVID risk; PPE access; PPE training and confidence; and anxiety. Participants were recruited from community and hospital healthcare settings in Australia, including doctors, nurses, allied health professionals, paramedics, and aged care and support staff. Data analysis was descriptive with free-text responses analysed using qualitative content analysis and multivariable analysis performed for predictors of confidence, bullying, staff furlough and anxiety. The 2258 respondents, comprised 80% women, 49% doctors and 40% nurses, based in hospital (39%) or community (57%) settings. Key findings indicated a lack of PPE training (20%), calls for fit testing, insufficient PPE (25%), reuse or extended use of PPE (47%); confusion about changing guidelines (48%) and workplace bullying over PPE (77%). An absence of in-person workplace PPE training was associated with lower confidence in using PPE (OR 0.21, 95%CI 0.12, 0.37) and a higher likelihood of workplace bullying (OR 1.43; 95% CI 1.00, 2.03) perhaps reflecting deficiencies in workplace culture. Deficiencies in PPE availability, access and training linking to workplace bullying, can have negative physical and psychological impacts on a female dominant workforce critical to business as usual operations and pandemic response.

## Introduction

Access to appropriate personal protective equipment (PPE) became a flashpoint for healthcare workers (HCWs) during the COVID-19 pandemic. At least 1.6 million HCWs have been infected as of 31 December 2020 [1], 17,000 HCWs are estimated to have died, and thousands more affected by chronic illness [2]. In Italy where at least 10% of HCWs became infected [3], 22% reported inadequate quality or quantity of PPE [4]. Severe shortages of PPE in the United States led to 87% of nurses re-using masks or respirators and 27% reporting exposure to COVID-19 patients without wearing any PPE [5], undoubtedly contributing to over 3,600 deaths [6]. PPE is regarded as the measure of last resort in the hierarchy of controls in healthcare, but it is important as higher order controls (i.e. elimination of hazards, substitution with telehealth, engineering changes or administrative measures) are often compromised or it is impossible for HCW to avoid the hazard when treating patients with COVD-19 is the job, rather than incidental to the job as in other industries [7]. Hence PPE may be the most important protection available and every element of the PPE ensemble matters as does the associated training.

Australia’s health system was ill prepared for the pandemic as indicated by escalating HCW infections during Victoria’s second wave. Total case numbers were 7767 until 30 June 2020 but increased to 19,080 by 31 August 2020, when Victoria experienced a resurgence in daily cases due to hotel quarantine breaches seeding into the community [8]. HCW infections spiked, increasing from 388 in Victoria on 16 July 2020 to 3580 by 31 December 2020 [9]. One HCW death was reported in April 2020 [10]. Of Victorian HCWs infected with COVID-19, 70% were occupationally acquired, 50% were aged care or disability workers, 40% were nurses or midwives, 4.8% were medical practitioners, and 5.7% were other types of HCWs [9].

COVID-19 infections remained relatively stable across Australia between January 2021– May 2021. Yet there has been a resurgence of cases in Australia, primarily in New South Wales, the country’s most populous state. As of 20 August 2021, there were 10,582 cases in NSW, largely attributable to the highly infectious Delta strain [11]. As of the 14^th^ of August, there have been 236 HCW infections in New South Wales since August 2020 [12], 53 (22%) were potentially infected in healthcare settings, 95 (40%) linked to social or household contacts and 88 (37%) cases were under investigation [13]. It was in this context that we surveyed HCWs around Australia about their experiences with PPE.

The main objectives of this study were to:

- Identify the use of PPE amongst HCWs including any barriers to training, access and workplace bullying over PPE.
- Examine the associations between HCW demographics (e.g. gender, years of experience and work setting) and anxiety, confidence with PPE, workplace bullying and experiences of COVID-19 restrictions).

## Methods

### Study design and participants

This was a national cross-sectional study conducted amongst HCWs including doctors (general practice, private physicians and hospital-based physicians and surgeons), nurses (community and hospital), allied health practitioners, paramedics and aged care workers in Australia.

### Survey development

An anonymous online survey of HCWs in Australia was developed by the research team who have extensive clinical and research experience in general medicine, infectious diseases, PPE, epidemics and survey methods fields. All survey questions and response options were written and reviewed closely by the team. We piloted the survey with two general practitioners to ensure that all functionality was intact and to refine the wording of questions and response options. The final survey consisted of a combination of multiple response options and open-ended questions that covered six domains including the GAD-7 scale [14] as shown in Table 1. Skip logics were used to reduce survey burden and responses were not forced due to the sensitive nature of the questions.

**Table 1:** Survey domains and variables.

Domain	Questions/Variables
**Demographics and employment:**	Gender Ethnicity Occupation Work setting and workplace characteristics State Postcode Hours worked
**Perception of COVID risk**:	Proportion of patients seen face to face in the past week Proportion of patients seen face to face before the pandemic Rating of risk of exposure (no risk, little risk, some risk or high risk) in March 2020 and Now (at the time of survey completion) Exposure at work requiring quarantine (yes/no) How many days in quarantine Tested for COVID-19 Mask wearing in public
**PPE training and confidence in use**:	Type of PPE training accessed (formal, in person, online) Who is best placed to provide PPE training (state health departments, federal government, royal colleges, primary health networks, individual workplaces, private training operator, other) Confidence in use and fitting of PPE (not at all confident, somewhat confident, mostly confident, very confident)
**Use and access to PPE:**	PPE items used prior to COVID-19 versus now (time of completion) (check list of PPE items for example face shield, P2/N95 mask, goggles) Additional forms of PPE (yes/no) Reasons not worn additional PPE (little risk, cannot access supplies, improvising alternative equipment, other) Barriers to accessing PPE supplies (lack of availability, workplace doesn’t supply PPE, high costs, delays in delivery, workplace ration supplies, other) Alternative equipment for PPE (garbage bags, homemade PPE, wearing scrubs, other) Reuse or extend the life of PPE (yes/no/do not use PPE) How reuse/extend life of PPE (e.g washing, disinfecting, heating, wearing for longer than recommended) Ranking the importance of PPE items in March versus now (time of completion) (e.g P2/N95 masks, eye and face protection, body protection, gloves, shoe covers, other) Ability to follow workplace PPE policy (no formal policy, unable to follow policy, partly, always) What prevented following PPE policy (e.g policy unclear, unable to access equipment, patient group unlikely to have COVID-19, not important, other) Have you ever been denied PPE when examining or treating a patient with suspected COVID-19 (yes, no)
**Workplace safety and culture**	Extent of experience of bullying (slider scale 0–100—I have experienced bullying and harassment, I have experienced coercion in the workplace over PPE, I have experienced ostracism and social inclusion in the workplace over PPE) Has a work health and safety professional assessed your workplace risk of COVID-19 (yes/no/don’t know) Has a work health and safety professional advised you on PPE (yes/no/don’t know) Has an infection control professional assessed your workplace risk of COVID-19 (yes/no/don’t know) Has an infection control professional advised you on PPE (yes/no/don’t know) Costs of PPE to workplace, cost of PPE personally Awareness of financial burden of PPE and telehealth/bulkbilling (sliding scale 0–100) Workplace needed to close due to lack of availability of PPE (yes permanently, yes temporarily, no) Satisfaction of advice on PPE from various groups (e.g employer, royal colleges, CDC, Australian Medical association–not at all satisfied, somewhat satisfied, mostly satisfied, very satisfied, not applicable)
**Anxiety**	Generalised Anxiety Disorder Scale (GAD-7). The GAD-7 is a brief measure for assessing symptoms of generalised anxiety disorder in the last two weeks. The response options for the 13 symptom items are “not at all,” “several days,” “more than half the days,” and “nearly every day,” scored as 0, 1, 2, and 3, respectively. The scale has been shown to have strong criterion, construct, factorial and procedural validity as well as good test-retest reliability. Scores of 5, 10 and 15 can be interpreted as mild, moderate and severe levels of anxiety [14].

### Recruitment strategy

Convenience sampling techniques were used. The survey advertisement and link was sent to professional organisations: Royal College of General Practitioners, HealthEd Pty Ltd (a primary care education company); the Australasian College of Paramedicine, Occupational Therapy Australia, the Victorian Ambulance Union, and Health and Community Services Union, the NSW Nurses and Midwives Association, the Queensland Nurses and Midwives’ Union, the Royal Australian and New Zealand College of Radiologists, the Victorian Perioperative Nursing Group and via social media channels including twitter and medical Facebook groups to capture a broad range of medical specialities from 1 July 2020 to 28 October 2020. As this study was exploratory in nature, a sample size calculation was not conducted. However, the sample size of previous cross-sectional studies amongst HCWs in China, Italy and Spain during the COVID-19 pandemic ranged from 59–5062 people [15]. As such, we anticipated that 2000 HCWs in Australia would participate in this survey.

### Data analysis

Data analysis was performed using Stata/IC 15.1 (StataCorp College Station, Texas, USA). Descriptive statistics were used to summarise the characteristics of all respondents which were reported using frequencies, means/medians, standard deviations/interquartile ranges as appropriate. The characteristics of respondents associated with outcomes including anxiety, confidence with PPE, bullying over PPE and experiences with COVID-19 restrictions were examined using multivariable logistic regression analyses. All variables were entered simultaneously into the multivariable model, and highly correlated variables were identified using the variance inflation factor (VIF) where values >3 indicated the presence of collinearity. Model findings were reported as odds ratio (OR) with 95% confidence intervals (CIs) and *p*<0.05 was considered statistically significant. Free-text responses to survey items where there was the option for ‘other’ and the final question *“Is there anything else that you would like to tell us about your experiences with PPE during COVID-19*?*”* were analysed using qualitative content analysis techniques. Two researchers (DB and CP) created a coding guide by deductively coding 20 responses together. The remaining open-ended responses were divided in two and coded separately using the coding guide. The codes were clustered into categories and further refined into themes. DA and MAR reviewed all codes, categories and themes.

### Ethics approval

Ethics approval was obtained from the Monash University Human Research Ethics Committee (Project ID 26132).

## Results

### Demographics and employment

Of 2258 respondents, 80% were female (*n* = 1856) and 79% (*n* = 1755) identified as Caucasian (Table 2). Doctors (*n* = 1141, 49%) and nurses (*n* = 924, 40%) predominated, followed by smaller numbers of allied health and non-clinical staff (*n* = 195, 8%). HCWs from both hospital (*n* = 899, 39%) and community (*n* = 1326, 57%) settings were represented. Most respondents were from Victoria (*n* = 939, 47%), New South Wales (*n* = 553, 28%) and Queensland (*n* = 271, 14%). The key survey quantitative and qualitative themes are described below. Table 3 presents the qualitative content analysis themes and illustrative quotes. The denominator across survey sections varied due to skip logics and non-forced responses.

**Table 2:** Demographic characteristics of HCW respondents.

Characteristic	All respondents (N = 2,258)
Gender	
Male	399 (17)
Female	1,856 (80)
Other (non-binary, transsexual, non-gendered)	3 (<1)
Ethnicity	
White	1,755 (79)
Non-white	466 (21)
Occupation	
Doctors	1,141 (49)
Nurse	924 (40)
Allied Health	121 (5)
Other (paramedic, personal care assistant, non-clinical staff)	74 (3)
Workplace type	
Hospital	899 (39)
Community	1,326 (57)
Other (Defence, education sector, custodial/justice, NGO)	30 (1)
State or Territory	
Victoria	939 (47)
New South Wales	553 (28)
Queensland	271 (14)
South Australia	98 (5)
Western Australia	90 (4)
Tasmania	21 (1)
Australian Capital Territory	22 (1)
Northern Territory	12 (<1)

HCW, health care worker; NGO, non-government organization.

All values presented as *n* (%) unless stated otherwise.

**Table 3:** Content analysis of open-ended responses throughout survey.

Code	Type of HCW	State	Corresponding Quotes
PPE training	Doctor working in the community	QLD	We all need training in correct donning and doffing
	Nurse working in a hospital	VIC	The anxiety about being redeployed to Covid ward as theatres were quiet, that was the only thing that fuelled anxiety, not knowing if you’re going to be sent and being responsible for correct infection control as being a spotter when we have only had 30 min instructions ourselves
	Doctor working in the community	VIC	As a GP, I have had zero training in PPE. In March, I had to very quickly learn everything about correct donning and doffing of PPE and taking of NP (*sic* nasopharyngeal) swabs myself from YouTube videos which were by nurses from other countries. At the time, I could not find any official Australian or otherwise training videos.
	Doctor working in the community	VIC	The staff at the aged care facility received personal training but it was assumed doctors like myself knew what to do even though I never had formal training except maybe many years ago when working in a hospital.
Fit-testing	Pharmacist working in the community	VIC	I haven’t had any face to face training or fit testing and I don’t think the masks fit me properly.
	Nurse working in a hospital	VIC	Despite my best efforts to advocate for correct fitting N95 masks and quantitative fit testing, my manager has stating that fit checking is at the responsibility of each nurse (and not the healthcare provider to their employees).
	Nurse working in a hospital	WA	No staff have been fit tested or provided access to N95. We have asked and been denied. This has contributed to staff worrying that if/when we have community transmission we will not be prepared or adequately protected.
	Doctor working in a hospital	VIC	The lack of fit testing for N95 (and lack of availability) is mind blowing.
Access to PPE (for example, hoarding PPE, rationing its use)	Doctor working in the community	NSW	It is provided to the group of practices run by our owner GP but was not getting dispersed to us as readily as his main practice. Was my initiative that started to access but gowns and goggles rationed significantly. Surgical masks unavailable unless considered high risk.
	Nurse working in a hospital	VIC	I feel like access was limited and made difficult by power hungry management team. I felt insulted by the insinuation that clinical staff were stealing.
	Doctor working in the community	VIC	Our local PHN (public health network) has been useless, zero support, zero information and very limited supplies which were delayed getting to us.
	Doctor working in the army	QLD	There was initial well founded concern that PPE would be stolen which resulted in a rationing mentality making PPE less accessible. There is frequent wastage with inappropriate PPE being supplied eg surgical masks with face shields being supplied to patients instead of staff. My workplace has a low tolerance for criticism so people tend to keep their thoughts to themselves.
Poor experience with PPE, including poor processes and re-use/extended use of PPE)	Doctor working in a hospital	NSW	The majority of the time when we have a patient being screened and isolated for COVID-19, the PPE put outside their room is inadequate and/or incorrect, with no rubbish bin for disposal and no hand sanitiser to wash hands. What’s the point?
	Nurse working in a hospital	VIC	We had nearly nothing during the first wave, we were keeping used gowns, N95 masks etc and salvaging every drop of hand sanitiser from bottles. It was far below the standards we would’ve liked for ourselves and our patients.
	Doctor working in the community	VIC	I’m dismayed at how unprepared Victoria was in terms of PPE stockpile. Our practice had to find our own PPE and wear them longer than we should and reuse masks so that we would have any PPE at all.
Inconsistent or contentious guidelines	Doctor working in the community	QLD	Conflicting advice about which mask to use & difficulty accessing N95 & surgical masks
	Midwife working in a hospital	NSW	It was frustrating receiving so many variations on what was appropriate for use of PPE, from my employer, the union, the state and federal government. This caused me much anxiety and stress during the initial stages of the pandemic.
	Nurse working in a hospital	VIC	Frustrating that rules varied significantly between different hospitals and workplaces.
	Nurse working in a hospital	Not stated	The DHHS guidelines have not kept up with growing evidence of airborne transmission. All organisations write their policies with reference to these guidelines. When challenged, all refer back to the DHHS and say ‘we are following the most current advice’. But when dealing with a Novel coronavirus we cannot be reliant on evidence as there is no time gather and study the evidence. There has been anecdotal evidence of airborne transmission from the outset. Preventative and cautious guidelines need to be adopted in this situation rather than the reactive policy that has resulted in such great numbers of HCW infections. Policy should also be nationally written so that individual health services are not interpreting or applying guidelines as suits them.
	Doctor working in a hospital	VIC	Workplace guidelines appear to constantly lag behind what should be standard.
Feeling deprioritised	Nurse working in a hospital	NSW	So many health workers getting infected, it seems the PPE does not work. How do we protect ourselves!
	Nurse working in a hospital	QLD	I am very concerned by the numbers of health professionals who have been infected by COVID in areas where COVID has significant community transmission. These people are being asked to risk death. . .Why is any level of transmission acceptable? Why are health care workers not supported to ensure they are not at risk of significant illness and/or long term effects and death.
	Doctor working in the community	VIC	General Practitioners totally forgotten by everyone and struggled enormously to get PPE. Still some struggling going on.
	Doctor working in the community	VIC	GPs have been largely ignored, everyone focuses on hospital workers as though we don’t count as frontline.
	Doctor working in a hospital	VIC	Health care workers have not been protected. The infection numbers confirm this.
	Doctor working in the community	QLD	Not taken seriously by employers, feel very under-supported in this area—profit before people!
Workplace bullying	Nurse working in a hospital	VIC	My employer has been providing non-medical grade N95 masks. I informed my managers, told to stop making trouble.
	Doctor working in a hospital	VIC	Bullying by the executive whenever PPE issues are raised is a huge problem.
	Doctor working in a hospital	VIC	Significant harassment, bullying, accusations, and coercion around PPE use occurred outside the workplace from colleagues from other healthcare services in response to me stating I was following the national, state, and organisation PPE guidelines.

### Perception of COVID risk

Perception of risk decreased over time with the 65.7% of 2248 respondents rating their risk of exposure to COVID-19 as ‘*some risk*’ or ‘*high risk*’ in March 2020 compared to 55.7% of 2224 respondents at the time of survey completion. The mean proportion of patients seen face to face in the last week of survey completion (n = 2168) dropped to 70% from 90% before the pandemic (n = 2204). Only 146 respondents of 2257 indicated that they had needed to quarantine with the average number of days of quarantine being nine (range 1–28). The majority of participants had been tested for COVID-19 (58.6%).

### PPE training and confidence in use

Of 2197 respondents who responded to the questions relating to PPE training, 20% did not receive any form of PPE training (*n* = 442). Just under half of the respondents (49%; n = 1082) accessed formal workplace training with 36% (n = 784) being in-person and 14% (n = 298) online. The remaining respondents accessed online training by a recognised provider (19%; n = 410) with a minority accessing YouTube (8%; n = 175). Respondents cited the need for “*more training early on and ongoing training”* (Nurse, state unknown). The lack of training *“fuelled anxiety*” (Nurse, VIC). Training quality was characterised by an “*inconsistent approach*” (Doctor, QLD).

Respondents stressed the importance of fit testing in protecting HCWs and were critical of the lack of interest shown by organisational leadership. Staff had “*asked and been denied*” fit tested respirators which had “*contributed to staff worrying that if/when we have community transmission we will not be prepared or adequately protected*.*”* (Nurse, WA). Respondents were unequivocal “*fit testing must become mandatory for nurses working on a covid ward*” (Nurse, state unknown).

### Use and access to PPE

With regards to accessing PPE, 251 of 2197 respondents were unable to access PPE, with the majority being community based HCWs. Barriers identified were supplier availability (53%; n = 133) and workplace rationing (53%; n = 135) followed by delivery delays (28%; n = 70), high costs (27%; n = 68), the workplace not supplying PPE (23%; n = 57) and denial of PPE based on a low perception of risk or inappropriateness by management (5%; n = 12). General practitioners (GPs) felt that they “*have been largely ignored*, *everyone focuses on hospital workers as though we don’t count as frontline*” (Doctor, VIC). Many were “*forced to buy products through alternative channels*…*and in some cases have paid up to triple the usual price*” (Doctor, SA) including going to “*Bunnings to get equipment for myself*” (Doctor, QLD) or resorting to making their own “*hand sewn gowns and head covers*” (Doctor, VIC). Offerings from the national stockpile were “*scandalous*” (Doctor, NSW), with one QLD doctor reporting they received six N95 masks from the stockpile after a request for supply “*one for each of our doctors- complete joke*!” (Doctor, QLD). Of concern 12% (216/1790) of respondents were denied appropriate PPE when caring for suspected COVID-19 due to supply issues (59%), rationing (23%) and a low perception of risk by management (10%).

Among 2131 respondents of 2197 responding to lack of access to PPE, 47% reported that they had to reuse or extend the life of their PPE with responses evenly spread between hospital and community settings at 47% and 46% respectively. “*We were keeping used gowns*, *N95 masks*, *etc*. *and salvaging every drop of hand sanitiser from bottles*.” (Nurse, VIC). A variety of methods were used to extend the life of PPE including: wearing items longer than recommended (30%), reusing single use disposable items (25%), disinfecting items with liquid cleaner (21%), washing (15%), air drying, (6%) and using sunshine (4%). The majority of respondents (96%) who reported reuse and extended use of PPE were metropolitan-based.

### Workplace safety and culture

Questions regarding workplace PPE policy were answered by 2084 participants of the 2258. About a third of these respondents (33%) were unable to follow workplace PPE guidelines due to a lack of a formal policy in 15%, with most of the latter being community based. Respondents complained about the frequent changes to PPE policy which contributed to bullying “*Constantly changing guidelines has created confusion and anxiety amongst staff and contributes to bullying behaviour over the use of PPE*.*”* (Nurse, state unknown). The quality of advice was questioned as “w*orkplace guidelines appear to constantly lag behind what should be standard*” (Doctor, VIC) and inconsistency highlighted with “*variations across healthcare networks which leads to staff working across networks implementing in Network X what Network Y are doing*” (Nurse, state unknown). Respondents believed that the *“delay in information from WHO regarding aerosol transmission was a huge fail”* (Doctor, WA) which meant the influential state-based “*DHHS guidelines have not kept up with growing evidence of airborne transmission*”. Precaution was emphasised because “*there is no time to gather and study the evidence*. *Preventative and cautious guidelines need to be adopted*…*rather than the reactive policy that has resulted in such great numbers of HCW infections*” (Nurse, state unknown).

Just over half (53%) the respondents answered questions about workplace bullying. Of the 1204 responses, 77% (n = 929) indicated that they had experienced bullying and harassment over PPE. “*Bullying by the executive whenever PPE issues are raised is a huge problem*” (Nurse, state unknown) with respondents told to “*stop making trouble*” (Nurse, VIC).

Seventy-eight percent of respondents completed the GAD-7 scale (n = 1753). Approximately 1 in 5 respondents (21.6%) showed moderate to severe anxiety in the past two weeks with almost half (48.5%) indicating some level of anxiety (mild, moderate or severe) in the past two weeks.

### Factors associated with anxiety, confidence with PPE, workplace bullying and staff furlough

After adjusting for socio-demographic characteristics, respondents in Tasmania appeared to have higher odds of being anxious but uncertainty was great (OR 3.43; 95% CI 1.14, 10.37) (Table 3). Those with less than 10 years of experience were more likely to report anxiety (OR 2.05; 95% CI 1.49, 2.82). Respondents in Western Australia (OR 0.38; 95% CI 0.21, 0.68) and those who did not receive in-person workplace PPE training were less likely to be confident with using PPE (OR 0.21; 95% CI 0.12, 0.37). In contrast, HCWs working in general practice (OR1.73, 95%CI 1.06, 2.84) and the community (OR 2.14; 95% CI 1.05, 4.36), as well as nurses (OR 2.10; 95% CI 1.23, 3.59) were more likely to report being confident with PPE use. HCWs who did not receive in-person workplace PPE training also had a higher likelihood of experiencing bullying over PPE use (OR 1.43; 95% CI 1.00, 2.03). However, allied health staff (OR 0.40; 95% CI 0.18, 0.90) and respondents in general practice (OR 0.22; 95% CI 0.13, 0.36) were less likely to experience bullying. A higher likelihood of being furloughed was associated with respondents in Victoria (OR 2.37; 95% CI 1.37, 4.10), those with less than 10 years’ experience (OR 1.88; 95% CI 1.15, 3.07) and full-time employment (OR 1.57; 95% CI 1.02, 2.41). Of note, PPE training was not associated with reduced anxiety or furloughing. Table 4 details the adjusted analysis.

**Table 4:** Characteristics of respondents associated with anxiety, confidence with PPE and experiences with COVID-19 restrictions and bullying over PPE: Adjusted odds ratio* with 95% confidence intervals.

	Anxiety	Confidence with PPE	Bullying over PPE	Experiences with COVID-19 restrictions
State New South Wales Australian Capital Territory Northern Territory Queensland South Australia Tasmania Victoria Western Australia	1 1.31 (0.36, 4.72) 0.65 (0.08, 5.37) 0.98 (0.62. 1.57) 0.83 (0.39, 1.80) **3.43 (1.14, 10.37)** 1.40 (1.00, 2.00) 1.00 (0.50, 1.99)	1 - - 1.11 (0.67, 1.84) 0.88 (0.43, 1.81) 0.37 (0.11, 1.25) 1.24 (0.83, 1.86) **0.38 (0.21, 0.68)**	1 0.57 (0.12, 2.66) 0.27 (0.05, 1.38) 1.14 (0.68, 1.92) 0.95 (0.46, 2.00) 3.73 (0.46, 30.12) 0.76 (0.52, 1.11) 1.14 (0.54, 2.41)	1 1.65 (0.21, 13.24) 2.65 (0.32, 22.30) 0.75 (0.31, 1.82) 0.30 (0.04, 2.33) - **2.37 (1.37, 4.10)** 1.73 (0.62, 4.83)
Work setting Hospitals General practice Community	1 0.75 (0.50, 1.15) 0.79 (0.48, 1.29)	1 **1.73 (1.06, 2.84)** **2.14 (1.05, 4.36)**	1 **0.22 (0.13, 0.36)** 0.75 (0.39, 1.44)	1 1.18 (0.62, 2.25) 1.40 (0.69, 2.86)
Sex Male Female	1 1.49 (1.00, 2.21)	1 0.90 (0.59, 1.38)	1 1.33 (0.89, 2.00)	1 1.03 (0.60, 1.79)
Ethnicity Caucasian Non-Caucasian	1 (1.05, 0.74, 1.49)	1 1.08 (0.73, 1.60)	1 1.34 (0.91, 1.98)	1 0.93 (0.55, 1.57)
Occupation Doctor Nurse Allied Health Other	1 1.44 (0.96, 2.18) 1.45, (0.73, 2.88) 2.81 (1.00, 7.87)	1 **2.10 (1.23, 3.59)** 1.16 (0.48. 2.81) 1.04 (0.22, 4.90)	1 0.83 (0.51, 1.37) **0.40 (0.18, 0.90)** 1.82 (0.37, 9.03)	1 0.95 (0.51, 1.76) 1.16 (0.44, 3.04) 0.62 (0.08, 4.83)
Years of experience ≥10 years <10 years	1 **2.05 (1.49, 2.82)**	1 0.72 (0.46, 1.12)	1 0.83 (0.55, 1.25)	1 **1.88 (1.15, 3.07)**
Employment status Part time Full time	1 0.95 (0.71, 1.26)	1 1.33 (0.93, 1.91)	1 1.33 (0.96, 1.85)	1 **1.57 (1.02, 2.41)**
PPE training received Workplace training in-person Formal training (not in-person) Alternate training No training	1 1.02 (0.72, 1.45) 1.43 (0.87, 2.35) 1.49 (0.99, 2.25)	1 **0.30 (0.17, 0.55)** **0.24 (0.12, 0.48)** **0.12 (0.06, 0.21)**	1 **1.71 (1.14, 2.56)** **1.80 (1.01, 3.19)** **1.82 (1.14, 2.92)**	1 1.09 (0.66, 1.83) 0.97 (0.43, 2.17) 0.98 (0.52, 1.87)

PPE, personal protective equipment.

*Multivariate models included state, work setting, sex, ethnicity, occupation, years of experience, employment status and PPE training received.

## Discussion

This study provides insights from the experience of frontline HCWs during the height of the second wave in Victoria last year, and holds relevance for current outbreaks in NSW and Victoria. The key findings reflected swiss cheese-like failures including inadequacies in PPE availability; training; fit testing of respirators; inconsistent guidelines and struggles with leadership which contributed to HCW infections. Strikingly, 80% of respondents were female reflecting the feminisation of the health workforce [16] where women have disproportionately borne the burden of COVID-19 both at home and at work [17]. Impacts included COVID-19 infections, discomfort, bullying, anxiety, financial costs, and a moral injury [18]. Inconsistent frequently changing guidelines that failed to recognise the airborne transmission of SARS-CoV-2, contributed to confusion and conflict with management, compromising care delivery under the added burden of PPE. The lack of bargaining power for many respondents limited their agency to resolve matters further exacerbating physical, psychological and financial impacts.

Despite the necessity of PPE training, a large proportion of respondents (47%) did not receive any formal training at work. This is in stark contrast to our Asian neighbours who have employed a variety of in-person training techniques including in-situ simulation, where high-stress clinical scenarios are role-played in order to improve real-world preparedness [19]. The provision of formal in-person training correlated with levels of confidence in respondents, while its absence correlated with workplace bullying perhaps reflecting broader deficiencies in workplace culture. The lack of in-person training was at odds with pre-pandemic national guidelines for infection control [20]. It is unclear why these guidelines were not enacted when the pandemic hit, especially when the consequences of incorrect PPE usage during a pandemic has increased significance. In fact, national infection control guidelines during the pandemic, downplayed fit testing because it “will be difficult due to limited supplies and range of types/sizes available” [21] only to be reinstated in 2021 in the aftermath of HCW infections and hotel quarantine leaks [22]. Severe shortages in PPE lead to rationing, private purchase and its reuse/extension by a variety of non-standardised methods including UV/sun light, heat and disinfection potentially putting HCWs in harm’s way [23].

The poor integration of modern work health and safety (WHS) principles in healthcare that was exposed nearly two decades ago by the Canadian SARS commission [24], remains pertinent today. Under Australian WHS legislation, an organisation is required to adopt reasonable and practicable controls such as the provision of fit tested respirators and appropriate parts of the PPE ensemble—when worker harm is likely, even when uncertainties around evidence prevail [7]. Although PPE is regarded as the least effective control, it must be strengthened especially when higher level controls (e.g. substitution, engineering, administrative) in healthcare settings are compromised or absent altogether [7]. Consultation between workers and management on matters relating to their safety is another legislative safeguard that was poorly executed as observed in other Australian [18] and international studies [25]. Notably, community-based respondents, more often lacking formal PPE policies, had a greater locus of control exhibiting decreased bullying and higher confidence. For example, general practitioners transformed their practice carparks into respiratory clinics [26] whereas hospital workers were bound by organisational policies and lacked agency to advocate for themselves. Junior HCWs were more likely to report anxiety or furlough reflecting their lower agency and higher risk of exposure in health care settings.

The occupational moral injury described by respondents echoed in an earlier study [18], could have far reaching effects. Moderate to severe anxiety was reported by more than one fifth (22%) of respondents, and several raised the prospect of leaving the healthcare sector. This threat should be taken seriously, as a health-care system cannot function in the long term by relying on the altruism and sense of duty in its workers [27]. A skilled workforce takes generations to develop and attrition of staff will have enduring effects.

A strength of this study is the high number of responses from community and hospital practice with the themes mirrored by another Australian study, providing external validity to our findings [11]. Limitations of this study include the lack of denominator data to gauge response to the survey. Specific groups like aged care workers and cleaners were under-represented but deserve targeted research given they comprised over 40% of HCW infections in Victoria [9]. Also underrepresented were nurses, allied health and people from culturally diverse backgrounds who may be less inclined to speak out about the issues explored in this study. To improve survey completion we used skip logics and did not force responses, however this led to changing denominators across questions, which may affect the generalisability and interpretation of the results.

Preventing infections in HCWs should be a national priority. They not only carry the burden of their personal health, but the potential to transmit COVID-19 to their co-workers, patients, family and the wider community. Apart from high quality PPE, in person training at work should be the minimum standard used for benchmarking because it correlates with greater confidence in using PPE and a better workplace culture.
